# Comparing self-assessment and instructor ratings: a study on communication and interviewing skills in psychology student training

**DOI:** 10.1186/s12909-025-06783-x

**Published:** 2025-02-11

**Authors:** Mats Najström, Martin Oscarsson, Ingrid Ljunggren, Jonas Ramnerö

**Affiliations:** 1 Department of Psychology, Stockholm University, Stockholm, Sweden; 2 Centre for Psychiatry Research, Karolinska Institute/Region Stockholm, Stockholm, Sweden

**Keywords:** Interviewing, Communication skills, Feedback, Self-assessment

## Abstract

**Background:**

A multitude of studies have investigated, assessed, and debated the merits of self-assessment within medical education and related fields, yielding inconclusive results. This raises questions about the reliability of self-assessment as a tool for evaluating competency development. The objective of the current study was to investigate the self-assessment accuracy of psychology students regarding their performance in training for interviewing and communication skills. A novel assessment instrument was employed for this purpose. The main research questions were: (1) How accurate are students’ self-assessments of their performance in comparison to instructors’ ratings? (2) How well do these self-assessments align with perceived changes in skill development at both group and individual levels?

**Methods:**

The study was conducted in three phases to achieve the research objective. In Phase 1, 206 psychology students from the first three semesters of a 5-year master’s program at Stockholm University conducted 15-minute video-recorded interviews, which were rated by instructors using an 11-item assessment instrument. Data were analyzed using Exploratory Factor Analysis (EFA). Phase 2 included 173 second semester students conducting 15-minute video interviews. These were reviewed in small groups, with both students and instructors rating the interviews using the same instrument. The process was repeated after one week. Two Confirmatory Factor Analyses (CFAs) were conducted on instructors’ and students’ ratings to validate the three-factor model identified in Phase 1. In Phase 3, correlation analyses and paired-samples t-tests were conducted at both group and individual levels to address the research questions.

**Results:**

The findings indicated high self-assessment accuracy, reflecting strong self-assessment abilities among the students. Comparisons between instructor ratings and students’ self-assessments of skill progression demonstrated good overall alignment. The validated assessment scale developed in Phases 1 and 2 shows potential for application in various educational contexts.

**Conclusions:**

This study demonstrates that students are capable of evaluating their own interviewing and communication skills and can take an active role in their skill development when provided with suitable tools and adequate training. The findings support the viability of self-assessment as an educational tool and suggest its integration into psychology training programs, emphasizing clear criteria and reflective practices.

## Introduction

In both the medical and psychological professions, interviewing and communication skills are widely regarded as core competencies [1, 2, 3]. This focus aligns with the belief that effective communication and the ability to establish strong relationships with patients are fundamental to a healthcare professional’s skill set. Training in these skills is integrated into the basic curriculum for many health professionals, or even mandatory, including professional psychologist training [4]. Evidence suggests that such training not only enhances patient satisfaction but also improves physicians’ empathy and self-efficacy while reducing physician burnout [5]. While the development of communication skills has been extensively studied among medical students [6, 7], social workers, and general clinical professionals [8, 9], there has been comparatively less research on this topic among psychology students [10]. Interviewing and communication skills include active listening, maintaining contact, empathy, and the ability to use open questions, as well as reflections and summations [11, 12, 13]. The underlying rationale for training these skills is, of course, that repeated practice should result in a progressive development. Video-feedback training has been a generally recommended method for learning interviewing and communication skills [14].

The skills of interest are typically assessed by instructors or independent raters, but there are examples of self-assessment [15]. While this form of assessment may primarily seem like an economical choice, reducing the time and effort for teaching staff in assessing interviews, self-assessment holds several attractive pedagogical features. It may promote motivation and guide students in their own learning and internalization of the criteria for competent performance [16]. These procedures may involve that students simply check answers and grade themselves on tests, but could equally well involve more complex strategies for monitoring one’s performance and identifying strategies for improving skills within a domain. Tiuraniemi and coworkers [15] found that self-rated interpersonal and communicational skills improved, in both psychology and medical students, even after a shorter period of training. However, the question remains: how accurate are students in assessing their own competence?

Numerous studies have examined, evaluated, and discussed the virtue of self-assessment in medical education and adjacent subjects [17]. In a review [18], it was concluded that previous studies have consistently demonstrated poor self-assessment accuracy when compared to expert ratings. However, the reliability claims of expert assessments as a gold standard are suspect, since inconsistency among expert raters is found across studies. In later studies comparing self-assessment to expert ratings in communication skills training specifically, results are conflicting. For instance, several studies have demonstrated a strong agreement between medical students’ self-assessments and instructors’ assessments [19, 20], while no significant correlations were observed between dental students’ self-assessments and faculty assessments [21]. Furthermore, when examining self-assessment accuracy of changes in communication skills before and after training among clinicians in a neonatal intensive care unit, it was found that the clinicians reported improvements in their self-assessments, which were not reflected in the expert evaluations [22]. No studies of self-assessment in psychology students were identified. These results raise questions about whether an attractive pedagogical feature may be dubious from an educational perspective, lacking the essential prerequisites of a trustworthy assessment of the development of competencies.

In an earlier study [10], we used an instrument, developed by Levitt [13] to assess various communication skills in psychology students during repeated training in interviewing and communication skills. The instructors rated video-taped interview segments in which students conducted interviews with different interviewees. Students’ performance was evaluated by the instructors on eleven items (see Table 1), on a Likert-type scale, with ratings from 1 (insufficient) to 4 (skillful). In the current study, this instrument was translated, validated, and adapted to align with the training format.

**Table 1:** Assessment instrument items

Item	Content
1. Attending	Demonstrating interest in, and focusing on, the client
2. Active listening	Demonstrating the ability to follow and understand the client
3. Reflecting on content	Clarifying, paraphrasing, and summarizing
4. Reflecting emotion	Demonstrating and communicating empathy by reflecting the client’s emotions
5. Probing/questioning	Demonstrating the use of purposeful questions to keep the session on track and encourage further communication
6. Challenging/confronting	Identifying discrepancies and having the ability to challenge or confront when necessary
7. Using non-verbals	Exhibiting appropriate use of body language, vocal tone, facial expressions, and eye contact
8. Opening the session	Opening smoothly and effectively
9. Closing the session	Closing smoothly and effectively
10. Immediacy	Using “I–you” statements and process-related questions when appropriate
11. Using silence	Allowing appropriate silence and demonstrating the ability to tolerate silence during the session

The objective of the current study was to investigate the self-assessment accuracy of psychology students regarding their performance in training for interviewing and communication skills. The study focused on the concordance between students’ self-assessments and instructors’ ratings, as well as the consistency of perceived changes in students’ performance at both group and individual levels. The main research questions were: (1) How accurate are students’ self-assessments of their performance in comparison to instructors’ ratings? (2) How well do these self-assessments align with perceived changes in skill development at both group and individual levels?

## Method

### Overall study design

To achieve the research objective, the study was conducted in three phases: the first phase involved identifying the internal structure between the different constituent skills in the assessment format; the second phase evaluated the validity of this structure; and the third phase addressed the two research questions. Data were collected as a part of a curriculum development project for the psychologist program at Stockholm University, a 5-year master’s program. Upon completion, and after one additional year of supervised practice, graduates can apply for a license to practice from the Swedish National Board of Health and Welfare.

### Instrument

The assessment format from Levitt’s study [13], as presented in Table 1, was translated and adapted to align with the training format of this study. The instrument comprises of 11 items rated on a four-point Likert-type scale ranging from 1 to 4, where 1– insufficient, 2– improving, 3– satisfactory, and 4– skillful. If a certain skill was not observed, it was marked N/A (not applicable), and coded 0.

### Phase 1: identifying the internal structure of the assessment instrument

#### Participants

Phase 1 of the study involved psychology students from the first three semesters of a 5-year master’s degree program at Stockholm University. A total of 206 students were recruited, consisting of 136 women and 70 men, with a mean age of 29.3 years (standard deviation [SD] = 8.10).

#### Study design

In phase 1, each participating student met with another person not known to him or her (generally another student doing “interview time” as a course requirement) for a 15-minute authentic interview on an everyday topic, that was perceived to be reasonably challenging and interesting but not provocative. The topics of the interviews were developed during seminars held prior to the interview sessions. Common topics included thoughts, feelings, and experiences related to life transitions, interpersonal relationships, and personal growth. The appropriateness of a topic was based on the students’ interests and the need for an interview scenario that required an affective bond between the student and the interviewee. This bond, in turn, necessitated appropriate session openings and closings, emotional reflection, use of non-verbal communication, and effective application of the other communication micro-skills of interest in this project. The students worked in groups, brainstorming potential topics, and ultimately selected one from their top three alternatives, in consultation with their instructor. All interviews were video-recorded. The video recordings were reviewed by an instructor and rated based on the eleven items described above in the assessment form.

To minimize potential inconsistency in the ratings among the instructors, who were all experienced clinical psychologists and teachers at the Department of Psychology, workshops were conducted prior to the start of the study. During these sessions, instructors watched interviews, performed assessments using the instrument employed in the study, and subsequently discussed their evaluations within the group. Each instructor participated in at least three such sessions. The workshops resulted in a document, detailing operationalized definitions of insufficient and skillful application of the eleven different micro-skills.

#### Statistical analyses

To analyze the internal structure and identify suitable factors in the assessment instrument, an Exploratory Factor Analysis (EFA) was conducted on data from instructors’ assessments of psychology students in the first three semesters (*N* = 206).

### Phase 2: validating the internal structure of the assessment instrument

#### Participants

Phase 2 of the study involved psychology students from the second semester of a 5-year master’s degree program at Stockholm University. This sample consisted of 173 individuals, 117 women and 56 men, with a mean age of 28.8 (*SD* = 7.90).

#### Study design

The same procedure as in phase 1 was followed, with the difference that the video recordings were watched and discussed in small groups, each comprising three to four students and an instructor, immediately after the interview. During these discussions, formative feedback was provided. This procedure was repeated one week later, following the same format. Feedback and suggestions from the first training session served as a means for students to improve their performance in the second session. Both instructor and the students independently rated the interviews using the eleven items of the assessment instrument. A document detailing operationalized definitions of insufficient and skillful application of the eleven micro-skills was shared with the students prior to their self-assessment.

#### Statistical analyses

In order to assess the validity of the 3-factor model identified through the EFA in phase 1, a Confirmatory Factor Analysis (CFA) was performed on data from instructors’ assessments of psychology students in the second semester. Additionally, the validity of the same model, as applied to students’ self-assessment, was evaluated using CFA.

### Phase 3: main research questions of self-assessment accuracy

#### Statistical analyses

Using data collected from students and instructors in phase 2, correlation analyses and paired samples t-tests were conducted at both group and individual levels to address the main objective of this study.

#### Data analysis

The EFA and CFA analyses were conducted using JASP Team (2023), JASP (Version 0.17.3). For the EFA, the Kaiser-Meyer-Olkin (KMO) measure of sampling adequacy was calculated, with a threshold value of ≥ 0.6 considered acceptable [23]. Bartlett’s Test of Sphericity (*p* <.05) was performed to confirm significant correlations among items [24]. Factors were extracted using principal axis factoring and retained based on eigenvalues ≥ 1. A direct oblim rotation was applied to allow for potential factor correlations. Internal consistency of the retained factors was assessed using Cronbach’s alpha, with values ≥ 0.7 considered acceptable [25]. For the CFA, model fit was evaluated using the following indices and thresholds [26]:

- CFI (Comparative Fit Index): ≥ 0.90 (good fit).
- RMSEA (Root Mean Square Error of Approximation): ≤ 0.06 (good fit).
- SRMR (Standardized Root Mean Square Residual): ≤ 0.08 (acceptable fit).
- Chi-Square Test: A non-significant result indicates good fit.

All other analyses were performed in IBM SPSS Statistics version 28 (IBM Corporation, Armonk, NY, USA).

## Results

To analyze the internal structure between the different constituent skills that were initially assessed by the instructors (*N* = 206), an EFA was performed. As a first step, the suitability of data for factor analysis was evaluated. The correlation matrix revealed the presence of many coefficients of 0.3 and above (Table 2). Bartlett’s Test of Sphericity [24] reached statistical significance (*p* <.001) and Kaiser-Meyer-Olkin value over 0.85 indicated good factorability, supporting the results of the correlation matrix.

**Table 2:** Correlation matrix for items in the assessment instrument

Item	1	2	3	4	5	6	7	8	9	10	11
1	–										
2	0.66**	–									
3	0.46*	0.55**	–								
4	0.42*	0.44*	0.47*	–							
5	0.54**	0.43*	0.49*	0.33*	–						
6	0.37*	0.24	0.38*	0.31	0.42*	–					
7	0.53**	0.41*	0.29	0.46*	0.41*	0.29	–				
8	− 0.36*	− 0.40*	− 0.23	− 0.18	− 0.30*	− 0.07	− 0.42*	–			
9	0.29	0.23	0.15	0.21	0.23	0.19	0.38*	− 0.33*	–		
10	0.11	0.14	0.21	0.25	0.07	0.11	0.16	− 0.15	0.04	–	
11	0.36*	0.34*	0.19	0.19	0.24	0.12	0.31*	− 0.29	0.25	0.22	–

Note. *N* = 206

*p <.05 **p <.01

The eleven items of the assessment instrument were then analyzed by means of an EFA based on parallel analysis [27] using Principal axis factoring and direct oblimin rotation. Cronbach’s alpha for the scale from the current sample was 0.714, which is acceptable and indicates good internal consistency of the items. The EFA identified three factors explaining 57.1% of the total variance. Items with loadings greater than 0.32 with the factor in question were used to characterize the factor solution [28]. Item 10 (Having immediacy) was excluded from the analysis, and subsequently, it was removed from the model due to its propensity to generate a distinct factor. The three factors were assigned descriptive labels as outlined below, with accompanying factor loadings provided for each item (see Table 3).

Cronbach’s alpha was calculated for each factor to assess the internal consistency reliability. The alpha values were as follows: Factor 1 = 0.80, Factor 2 = 0.71, and Factor 3 = 0.69. The values for Factor 1 and 2 indicate satisfactory reliability, while the value for Factor 3 falls below the typical 0.70 threshold. However, given the good interrelatedness of the items in this factor and their solid loadings onto a single factor, the alpha value is considered acceptable [29]. Two-item factors are not ideal; however, as an additional measure of reliability for Factor 1 the Spearman-Brown formula was applied, yielding a coefficient of 0.80. This value meets the commonly accepted threshold of 0.70, supporting the inclusion of the two-item factor [30]. Additionally, item 2 demonstrates a very high factor loading (0.89) and a strong correlation with item 1 (*r* =.66). To accept the two items as a subscale/factor is consistent with the reasoning by Worthington & Whittaker [31] who argue that two items can be acceptable for forming a subscale/factor when they exhibit strong loadings and a substantial inter-item correlation.

**Table 3:** Results from an EFA with instructor rating data

	Factor loading		
	1	2	3
Factor 1: Attention			
1. Attending	0.40		
2. Active listening	0.89		
Factor 2: Reflection			
3. Reflecting on content		0.50	
4. Reflecting emotion		0.40	
5. Probing/questioning		0.51	
6. Challenging/confronting		0.66	
Factor 3: Structure			
7. Using non-verbals			0.54
8. Opening session			0.59
9. Closing session			0.57
11. Using silence			0.44

Note. *N* = 206

A CFA was performed to assess the validity of the previously mentioned three-factor model, utilizing instructor rating data collected from second-semester psychology students (*N* = 173). The chi-square test indicated a significant difference between the observed and estimated covariance matrices (χ² (32) = 55.21, *p* <.01). However, it is important to acknowledge that this test is sensitive to sample size. Given that the other fit indices consistently indicate that the observed data align well with the model, a comprehensive evaluation supports the assertion that the 3-factor model is a valid and reliable measure of instructors´ assessment of the students’ performance. Information regarding respective factor loadings is found in Table 4 and fit indices in Table 5.

**Table 4:** Results from a CFA with instructor rating data

	Factor loading		
	1	2	3
Factor 1: Attention			
1. Attending	0.44		
2. Active listening	0.52		
Factor 2: Reflection			
3. Reflecting on content		0.52	
4. Reflecting emotion		0.43	
5. Probing/questioning		0.39	
6. Challenging/confronting		0.35	
Factor 3: Structure			
7. Using non-verbals			0.38
8. Opening session			0.36
9. Closing session			0.42
11. Using silence			0.34

Note. *N* = 173

**Table 5:** Goodness-of-fit indices for three-factor model

Indices	Value	Model Fit Decision
CFI	0.96	good
RMSEA	0.06	acceptable
SRMR	0.05	good

An additional CFA was performed to assess the validity of the aforementioned three-factor model when applied to the students´ self-assessment data (*N* = 173). The chi-square test indicated a significant difference between the observed and estimated covariance matrices (χ²(32) = 51.32, *p* =.02). In accordance with earlier reasoning, a comprehensive assessment, including fit indices, indicates a favorable alignment between the observed data and the three-factor model. This suggests that the three-factor model serves as a valid and reliable measure for evaluating students’ self-assessment. Information regarding respective factor loadings is found in Table 6 and fit indices in Table 7.

**Table 6:** Results from a CFA with student self-assessment data

	Factor loading		
	1	2	3
Factor 1: Attention			
1. Attending	0.48		
2. Active listening	0.53		
Factor 2: Reflection			
3. Reflecting on content		0.52	
4. Reflecting emotion		0.43	
5. Probing/questioning		0.45	
6. Challenging/confronting		0.58	
Factor 3: Structure			
7. Using non-verbals			0.48
8. opening session			0.46
9. Closing session			0.30
11. Using silence			0.54

Note. *N* = 173

**Table 7:** Goodness-of-fit indices for three-factor model

Indices	Value	Model Fit Decision
CFI	0.96	Good
RMSEA	0.06	Acceptable
SRMR	0.05	Good

Descriptive statistics for instructors´ ratings and students’ self-assessments regarding the overall measure and the three factors on the two separate training sessions are presented in Table 8.

**Table 8:** Mean values and Standard Deviation (SD) for overall scoring of each factor and training session

	Training 1		Training 2	
	Students	Instructors	Students	Instructors
Overall	26.00 (5.12)	26.19 (4.08)	28.86 (5.08)	28.87 (4.41)
Factor 1	5.44 (1.51)	5.84 (1.11)	6.26 (1.19)	6.27 (1.16)
Factor 2	9.97 (2.12)	9.77 (2.25)	10.71 (2.41)	10.96 (2.41)
Factor 3	10.60 (2.42)	10.57 (1.85)	11.89 (2.18)	11.64 (1.73)

Note. *N* = 173

A paired-samples *t*-test was conducted to examine self-assessment accuracy across the two training sessions. The results revealed a significant difference between instructors’ and students’ scores on factor 1 during training session one (*t*(172) = 3.15, *p* =.002) corresponding to a small effect size Cohen’s *d* = 0.24 [32]. No other statistically significant findings were observed.

The descriptive statistics for the progression in instructors’ ratings and students’ self-assessments regarding the overall measure and the three factors from training session one to training session two are provided in Table 9.

**Table 9:** Mean values and Standard Deviation (SD) for progression in overall scoring and each factor

	Progression	
	Students	Instructors
Overall	2.86 (5.74)	2.68 (4.89)
Factor 1	0.82 (1.77)	0.43 (1.42)
Factor 2	0.75 (2.39)	1.18 (2.61)
Factor 3	1.29 (2.62)	1.06 (2.27)

Note. *N* = 173

To assess the students’ self-assessment accuracy of progression regarding the overall measure and the three factors from training session one to training session two, a paired-samples *t*-test was conducted. The analysis revealed a single statistically significant result: students’ self-assessment increased significantly more compared to instructors’ rating on Factor 1, (*t*(172) = 2.60, *p* =.01) corresponding to a moderate effect size (*d* = 0.47).

To examine the congruence between instructors’ assessments and students’ self-assessments, a correlation analysis was conducted to investigate the relationship between the change in scoring from training session one to training session two for both groups. The results are presented in Table 10.

**Table 10:** Correlation for change in overall scoring and each factor

Assessment pair	Correlation
1. Student overall - Instructor overall	0.74**
3. Student factor 1 - Instructor factor 1	0.51**
4. Student factor 2 - Instructor factor 2	0.40**
5. Student factor 3 - Instructor factor 3	0.32**

Note. *N* = 173

**p <.01

## Discussion

The main objective of this study was to investigate the self-assessment accuracy of psychology students regarding their performance in training for interviewing and communication skills, an area that has been understudied compared to other health professions. We were particularly interested in examining the concordance between student and instructor ratings, as well as the correspondence of perceived changes in performance at both the group and individual levels. The latter suggests an ability to recognize skill development throughout the training process.

To address this main objective, Phases 1 and 2 of the study were conducted to identify and validate the internal structure of the assessment instrument. Our findings revealed a high degree of homogeneity, and the results from the EFA indicated a three-factor model as the underlying construct. The factors were labelled as follows: Factor 1: Attention, Factor 2: Reflection, and Factor 3: Structure. The subsequent CFAs demonstrated good validity for the model when applied to the instructors’ and students’ data, respectively. This is in accordance with prior research indicating that the assessment of communication skills can be dismantled into distinct factors [33, 34, 35, 36].

In relation to research questions (1): “How accurate are students’ self-assessments of their performance in comparison to instructors’ ratings?”, the overall findings suggest a high level of self-assessment accuracy across both training sessions, including both aggregate scores and individual factors. These findings are in line with those in previous research [19, 20], while there are also contradicting results [21]. Notably, a significant difference emerged in the factor Attention during training session one, where students, as a group, undervalued their performance in comparison to instructors’ ratings. However, this disparity was not evident in assessments of performance in training session two. One possible explanation for this finding lies in the analysis of the progression from training session one to two, revealing a significant difference between changes in instructor and student ratings concerning the factor Attention. Students seem to have overestimated their advancement in this factor compared to instructors, resulting in a convergence of scores at session two. Aside from this difference in factor Attention during session one, there was good self-assessment accuracy in session one and session two, both in the overall measure and in the factors of Reflection and Structure.

When examining students’ self-assessment accuracy of progression from session 1 to session 2 in relation to research question (2), we found strong agreement at the individual level for both the overall measure and the separate factors of Attention, Reflection, and Structure. The significant correlations indicate a strong alignment between students’ and instructors’ evaluations of the development of communication and interviewing skills. These findings contrast with a study in which clinicians working in a neonatal intensive care unit reported improvements in their communication skills that were not corroborated by expert evaluations [22]. However, our results suggest that repeated training enhances proficiency, consistent with the reasoning of Tiuraniemi and colleagues [15]. Furthermore, self-assessment appears to highlight both weaknesses and strengths for students, suggesting that their self-perception may become more accurate over time. More importantly, it accentuates that self-assessment may be a viable alternative as an educational model, optimizing resource utilization. As a result of Phases 1 and 2 of this study, an assessment scale has been validated and can be utilized in various ways. For instance, instructors can use the scale to systematically evaluate students’ performance in specific skills. Students can use it to reflect on and assess their own progress, as well as identify areas in need of improvement. Additionally, the scale can serve as a tool for providing detailed and structured feedback. Instructors can use it to explain the scores and offer concrete examples of how students can improve their performance in each category.

In this study, we argue that high self-assessment accuracy reflects strong self-assessment ability. This assumption is based on the premise that the instructor’s rating serves as the gold standard for measuring communication and interviewing skills. However, as noted in an earlier study [18], both the reliability and validity of this gold standard are subject to skepticism.

A pedagogical implication of this study is its advocacy for the thoughtful integration of self-assessment into psychology education. Instructors can enhance self-assessment accuracy by providing clear criteria, fostering reflective practices, and incorporating structured feedback sessions. These pedagogical strategies may empower students to actively engage in their skill development, aligning with the reasoning presented in earlier studies [16].

This study had some notable limitations. The design included only 2 training sessions with a one-week interval. Ideally, there should have been additional sessions to allow for a more thorough evaluation of the efficacy of repeated training. The study was conducted on a sample of psychology students, which limits the generalizability of the results. The same applies to the assessment instrument used. One potential limitation of this study is the inclusion of a two-item factor, which may not provide the depth typically required for robust construct representation. To address this concern, an additional reliability analysis was conducted, which provided support for retaining the factor. Furthermore, the two items capture a distinct aspect of the underlying construct that is not measured by any other items. According to the reasoning of Worthington and Whittaker [31], a two-item factor can be acceptable when the items demonstrate strong statistical and theoretical support. In this study, Item 2 shows a very high factor loading and a strong correlation with Item 1, further justifying the retention of the two-item factor. Another potential limitation is that the students did not receive prior training on how to use the self-assessment format. However, the self-assessment accuracy was good during the first training session, suggesting that the students accurately interpreted the different items.

## Conclusion and future directions

The study provides valuable insights into the self-assessment of communication and interviewing skills among psychology students, highlighting the interplay between training effectiveness, self-assessment validity, and the nuanced nature of various communicational skills. The findings contribute to the ongoing dialogue on the viability of self-assessment in educational settings, emphasizing the importance of context-specific factors and the need for continued research to refine assessment practices. Moreover, this study demonstrates that students can accurately assess their own communication skills when provided with appropriate tools and effective training. The results indicate high self-assessment accuracy, both for individual training sessions and for skill progression from the first to the second session. Future research could explore whether the observed agreement between self-assessments and instructors’ ratings persists across multiple training sessions and over longer timeframes. Longitudinal studies may reveal whether students’ self-assessment accuracy improves further as they gain more experience or whether potential biases emerge with increased familiarity with the training context. Additionally, further research is needed to critically evaluate the instructors’ ratings as the so-called ‘gold standard,’ addressing potential limitations in reliability and validity that may influence the alignment between self-assessments and external evaluations. Another important yet often overlooked perspective in the training process is that of the interviewees. Future research could examine how interviewees perceive the interview process, their interactions with student interviewers, and how these perceptions may inform both training approaches and assessment criteria. Incorporating interviewee feedback could provide a more holistic understanding of the communication and interviewing skills being developed, offering new dimensions to the evaluation framework. In conclusion, this study underscores the value of self-assessment as an educational tool, demonstrating its potential to empower students to take an active role in their own learning and skill development. By refining assessment practices and integrating perspectives from all stakeholders, future research can further enhance the utility and validity of self-assessment in psychology training and beyond.

## Data Availability

The datasets used during the current study are available from corresponding author upon request.

## Abbreviations

CFA: Confirmatory Factor Analysis
CFI: Comparative Fit Index
EFA: Exploratory Factor Analysis
KMO: Kaiser–Meyer–Olkin measure
RMSEA: Root Mean Square Error of Approximation
SRMR: Standardized Root Mean Square Residual
